# Tumor Heterogeneity of STEAP4 in Malignant Progression of Oral Squamous Cell Carcinoma

**DOI:** 10.7150/jca.101470

**Published:** 2024-11-04

**Authors:** Zheng Wu, Wen-Jia Chen, Yang-Zheng Lan, Ze-Xuan Fang, Yan-Yu Hou, Xin-Ning Yu, Hua-Tao Wu, Jing Liu

**Affiliations:** 1 The Breast Center, Cancer Hospital of Shantou University Medical College, Shantou 515041, China.; 2 Department of Physiology, Shantou University Medical College, Shantou 515041, China.; 3 Department of General Surgery, the First Affiliated Hospital of Shantou University Medical College, Shantou 515041, China.

**Keywords:** STEAP4, oral squamous cell carcinoma, biomarker, heterogeneity

## Abstract

**Background:** Recent research suggests that STEAP4, a metalloreductase *in vivo*, plays a crucial role in various types of tumorigeneses, especially in gastrointestinal cancers. However, few oncogenes have been reported in oral squamous cell carcinoma (OSCC). Therefore, this study aimed to explore the potential role of STEAP4 in OSCC.

**Methods:** The expression level of STEAP4 in OSCC tissues and adjacent normal tissues, was detected using immunohistochemistry. Publicly available online tools were utilized to analyze the expression, prognostic significance, and related enriched pathways of STEAP4 in head and neck squamous cell carcinoma (HNSCC) and OSCC. The relationship between STEAP4 expression and clinicopathological parameters in OSCC patients was validated using the χ2 test and Fisher's exact probability test.

**Results:** STEAP4 exhibited low expression in both HNSCC and OSCC. Whereas the prognosis for HNSCC patients was favorable, OSCC patients had poor outcomes. Genetic variability analysis revealed no alterations in STEAP4 in OSCC, whereas gene amplification was observed in HNSCC, suggesting tumor heterogeneity in STEAP4 among these cancer types.

**Conclusion:** STEAP4, as a risk factor associated with poor patient prognosis, shows tumor heterogeneity in OSCC patients, that is potentially related to genetic mutations or differences in histological distribution of oral mucosa. These findings indicate that STEAP4 could serve as an independent predictor for assessing the prognosis of OSCC patients.

## Introduction

Six-transmembrane epithelial antigen of prostate 4 (STEAP4), also known as six-transmembrane protein of prostate 2 (STAMP2) and TNFα-induced adipose-associated protein (TNFAIP9), belongs to the prostate six-transmembrane epithelial antigen of prostate (STEAP) family, which has significant roles in regulating glucose metabolism, fatty acid metabolism, and inflammatory responses^1^-^3^. During a gene product screen of TNF-α-induced pre-differentiated adipocytes, Korkmaz *et al.* initially identified and reported STEAP4 in 2012^1^. It is observed that STEAP4 co-localizes with transferrin and transferrin receptor 1 in the Golgi complex and plasma membrane^4^,^5^. Soon, numerous studies have indicated a correlation between STEAP4 and obesity, as well as insulin resistance^6^,^7^. Orfanou *et al.* suggested a potential anti-apoptotic role of STEAP4 in breast cancer^8^. Furthermore, STEAP4 has been implicated in inflammatory arthritis through its regulation of inflammatory cytokines^3^, and it also modulates the progression of androgen-dependent prostate cancer cells by inhibiting anchorage-dependent cell proliferation^9^.

On the other hand, oral squamous cell carcinoma (OSCC) is a prevalent malignant tumor, accounting for approximately 90% of all oral malignancies^10^,^11^. According to data from the Global Cancer Observatory (GCO), it was projected that by 2020, there would be 377,713 cases of OSCC worldwide, with a majority of cases occurring in Asia^12^. Moreover, the incidence of OSCC is expected to rise by approximately 40% by 2040, accompanied with an increase in mortality^12^. Extensive research has identified several risk factors contributing to the development of OSCC, including persistent exposure to smoking^13^, chronic alcohol abuse^14^, and chronic betel nut chewing^15^. Additionally, repeated infection with human papillomavirus (HPV) has been associated with the development of oral potentially malignant diseases (OPMDs)^16^, such as oral leukoplakia (OL), oral erythema (OE), oral submucous fibrosis (OSMF), and oral lichen planus (OLP). Among these OPMDs, proliferative verrucous mucosal leukoplakia (PVL) is a rare multifocal OL characterized by higher aggressiveness and recurrence rates^17^. OE, another malignant lesion, exhibits a higher potential for malignant transformation, with approximately 50% of patients progressing to atypical hyperplasia, carcinoma *in situ*, or invasive tumors^18^. Although various therapeutic interventions, such as chemotherapy, radiotherapy, immunotherapy, and nanomedicines, have been proposed for OPMDs or OSCC, unraveling the oncogenes and related biomarkers specific to OSCC can significantly contribute to early detection, diagnosis, and treatment of this malignancy, thereby improving the survival and prognosis of OSCC patients.

In this study, we investigated the expression and prognostic value of STEAP4 in OSCC. Additionally, we analyzed the clinicopathological parameters, associated pathways, and prognostic significance of STEAP4 in both HNSCC and OSCC using bioinformatics analytical tools and immunohistochemical staining. Our findings unveiled the tumor heterogeneity of STEAP4 in OSCC, highlighting its potential as an independent predictor of OSCC prognosis.

## Materials & Methods

### Patient information and ethical statement

A tissue microarray with 44 cases of OSCC tissues and 4 corresponding adjacent normal tissues were obtained for evaluating the expression level of STEAP4 by immunohistochemistry. This study was approved by the Ethics Committee of Shantou University Medical College.

### Molecular Structure and Function Prediction of STEAP4

The 3D structure of STEAP4 was predicted using the Swiss-Model website, which utilizes homology modeling (https://swissmodel.expasy.org/). GO database annotation information for the STEAP4 protein (Q687X5) was obtained from the Uniprot database (https://www.uniprot.org/) for functional classification. The STRING database (https://cn.string-db.org/) was used to predict the protein-protein interaction networks (PPIs) of STEAP4, mining the core regulatory networks associated with STEAP4-related genes.

### Expression of STEAP4 in different tissues and organs

STEAP4 expression in various tissues and organs was analyzed using the Expression Atlas (https://www.ebi.ac.uk/gxa/about.html), which provides information on gene expression patterns under different biological conditions, including gene knockout and treatment with specific compounds, containing information on microarray and RNA-seq data. Additionally, STEAP4 expression in HNSCC and adjacent normal tissues was analyzed using the GTEx (The Genotype-Tissue Expression) database and the PCAWG (The Pan-Cancer Analysis of Whole Genomes project) database. Bgee (https://www.bgee.org/) retrieved expression and deletion data for STEAP4 across different tissues and organs by integrating multiple data types (RNA-Seq, Affymetrix, *in situ* hybridization, and EST data). Data were derived from the GTEx dataset and other smaller datasets with consistent data annotation and processing.

### Expression of STEAP4 mRNA in HNSCC and prognostic analysis

STEAP4 expression levels in HNSCC were predicted using the ENCORI database (https://rnasysu.com/encori/). The UALCAN portal (https://ualcan.path.uab.edu/) was used to analyze the differential expression of STEAP4 mRNA in HNSCC patients and its relationship to tumor staging from a cohort of TCGA, including 520 primary HNSCC and 44 normal samples.

The PanCanSurvPlot (http://zjyy-oncology.asuscomm.cn:20008/) website was used to predict the overall survival (OS) for STEAP4 in HNSCC patients. Relapse-free survival (RFS) information was evaluated through PrognoScan (http://dna00.bio.kyutech.ac.jp/PrognoScan/). The OShnscc (https://bioinfo.henu.edu.cn/HNSC/HNSC_TCGA.jsp) website, integrating gene expression profiles of 1366 unique HNSCC cases and corresponding clinical information from nine independent cohorts, was used to predict the prognostic value of STEAP4 in TCGA and three GEO datasets (GSE31056, GSE65858, and GSE3292). Univariate Cox regression analysis was performed to show the role of STEAP4 in OS and disease-free survival (DFS) of patients, with hazard ratios (HRs) and their 95% confidence intervals, and log-rank *p-*values.

### Differential expression analysis and enrichment analysis in normal and OSCC tissue

Differentially-expressed genes (DEGs) in normal and OSCC tissues in the GSE31056 dataset were analyzed using the GEO database (https://www.ncbi.nlm.nih.gov/geo/). GO and KEGG enrichment analyses of DEGs was performed using R package.

### Expression of STEAP4 mRNA in OSCC and prognostic analysis

STEAP4 expression in DEGs was plotted as a bar graph. The "survival" algorithm of R package was used to calculate the survival of patients in the GSE31056 and GSE27020 datasets, with high and low STEAP4 expression, aiding in evaluating patient prognosis.

### Immunohistochemical staining and scoring

Tissue samples underwent deparaffinization in xylene, hydration in graded ethanol, antigenic epitope retrieval in ethylenediaminetetraacetic acid, and blocking of endogenous peroxidase in 3% H_2_O_2_. Samples were incubated overnight with an anti-STEAP4 antibody (Proteintech 11944-AP) diluted at 1:400, followed by hematoxylin nuclear re-staining for visualization. Sections were independently observed and evaluated under a bright-field microscope by two individual investigators blinded to patient information. STEAP4 expression was evaluated based on staining intensity (0-3: background color, light yellow, brown, and dark color) and the percentage of positive cells (0-4: 0%, 1%-25%, 26%-50%, 51%-74%, 76%-100%). Patients were categorized into low and high expression groups based on the total score results^19^. The relationship between STEAP4 level and clinicopathological parameters of OSCC patients was analyzed using the χ2 test and Fisher's exact probability test.

### Mutations of STEAP4 in HNSCC and OSCC

Genetic STEAP4 alterations in HNSCC were studied using the HNSCC-TCGA PanCancer Atlas in the cBioPortal platform (https://www.cbioportal.org/)^20^, which includes 523 samples. Genetic alterations in STEAP4 in OSCC were analyzed using 40 samples from the OSCC dataset (MD Anderson Cancer Discov 2013).

## Results

### STEAP4 plays dual roles as an oncogene and anti-oncogene in various types of cancers

**Figure 1:**
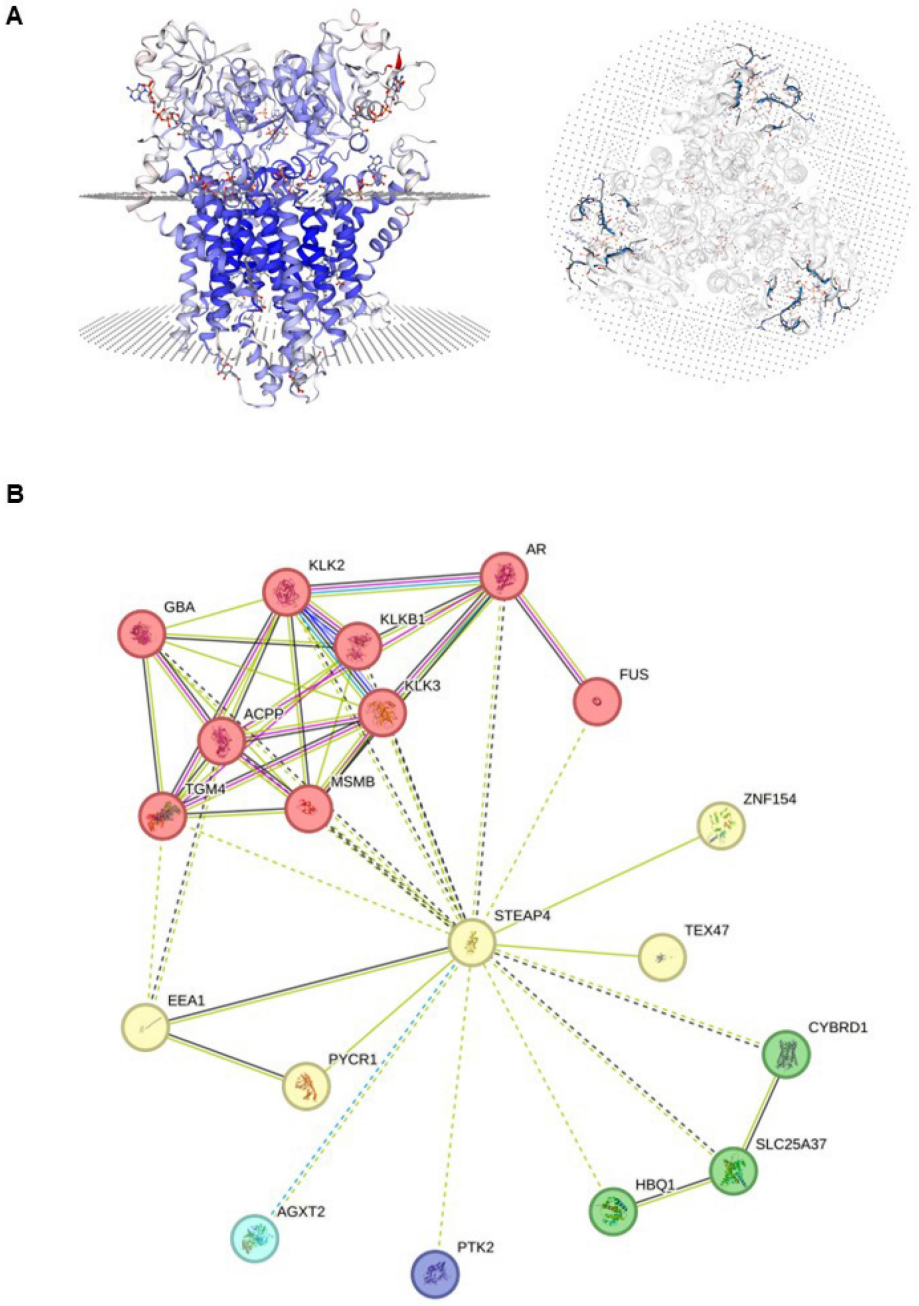
** Characteristics of STEAP4 protein.** (A) The structure of human STEAP4 from Swiss-Model. (B) Protein-protein interaction (PPI) network of STEAP4. The proteins interacting with STEAP4 were analyzed by k-means clustering that determines the number of proteins based on central points. The red protein cluster indicates mixed protein such as β-trace amino protein and prostate adenocarcinoma. The yellow protein cluster represents protein associated with cupric reductase activity and mitotic spindle midzone. The green protein cluster includes CYBRD1, HBQ1, SLC25A37; The blue protein cluster includes AGXT2; The purple protein cluster includes PTK2, which indicates different protein clusters with unknown function.

STEAP4 is a protein-coding gene that belongs to the family of STEAP and is situated on chromosome 7q21, comprising five exons and four introns. As shown in Figure 1A, STEAP4 is a transmembrane protein made up of 459 amino acids (52.0 kDa), possessing three conserved motifs in the N-terminal structural domain. These motifs correspond to a dinucleotide-binding structural domain, an NADP oxidoreductase motif, and a pyrrolidine-5-carboxylic acid reductase-like motif, with six transmembrane regions near the COOH-terminal domain^1^.

As an NADPH-dependent chelating iron-reductase, STEAP4 is found to be located in the Golgi^4^,^5^ and can reduce Fe(3+) to Fe(2+) using NAD(+) as an acceptor, which mediates a sequential transmembrane electron transfer from NADPH to FAD, to heme, and finally to Fe(3+) chelates^21^. Additionally, STEAP4 also reduces Cu(2+) to Cu(1+). GO annotations related to this gene reveal that the cellular components related to STEAP4 are mainly the extracellular exosome, endosome, membrane, and nucleoplasm. The molecular functions of STEAP4 are mainly focused on cupric reductase activity, metal ion binding, and electron transfer activity. STEAP4 also serves to maintain cupric iron homeostasis and assist electron transfer (Table 1).

Recently, there have been continuous discoveries regarding the function of STEAP4 in various types of cancer. When comparing different tissues and organs, the expression of STEAP4 mRNA and protein was found to be higher in adipose tissues, bone marrow, and prostate, while lower in brain, renal pelvis, and thymus (Supplementary Table 1). Among various diseases, the expression of STEAP4 was found to be elevated in luminal breast tumor and colon mucinous adenocarcinoma, but decreased in chronic lymphocytic leukemia, HNSCC, and breast adenocarcinoma (Supplementary Table 2).

Analysis of PPI networks revealed a close relationship between STEAP4 and the kallikrein-related peptidase (KLK) family, a serine protease family categorized under protein hydrolases (Figure 1B). It has been reported that the KLK family has varying effects on different components of the tumor microenvironment^22^. KLK3 is widely used as a PSA in the clinic^23^, and KLK4 and KLK5 are involved in the treatment and prognosis of other cancers, such as ovarian cancers 24, triple-negative breast cancers (TNBC)^25^ and colorectal cancers (CRC)^26^. These findings suggest that STEAP4 may exert biological effects through the KLK family, but this conclusion requires further validation.

In summary, STEAP4 exhibits differential expression in various tissues and can function as either a promoter or suppressor of cancer in different cancer types. Therefore, we were interested in exploring the expression of STEAP4 in HNSCC, especially in OSCC, and subsequently conducted further investigations and validations.

### STEAP4 serves as a protective factor in HNSCC

**Figure 2:**
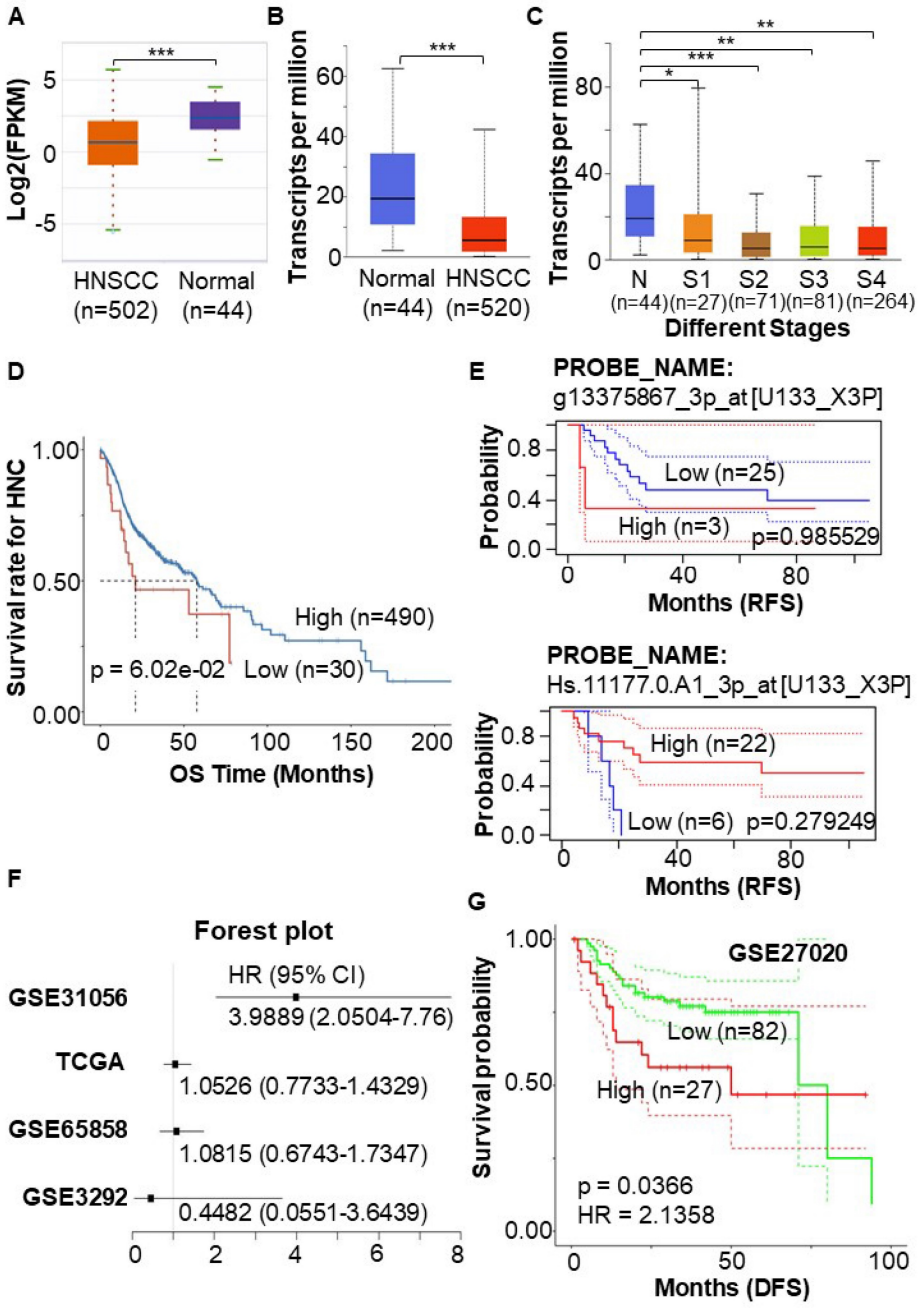
** Expression pattern and prognostic value of STEAP4 in HNSCC.** (A & B & C) Expression of STEAP4 in HNSCC in ENCORI (A) and UALCAN (B & C). (D & E) OS and RFS of STEAP4 levels for patients with HNC (D) and HNSCC (E). (F) Correlation of STEAP4 with OS. (G) DFS for STEAP4 levels for patients with HNSCC in GSE27020. Student's *t*-test was used to estimate the significance of the differences in gene expression levels between groups. * indicates *p* < 0.05, ** indicates *p* < 0.01, and *** indicates *p* < 0.001. HR (Hazard Ratio) was calculated in survival analysis data to estimate the risk ratio of death/remission/recurrence due to the presence of a certain factor.

To validate the lower expression of STEAP4 in HNSCC compared to adjacent normal tissues, regardless of HPV positivity^27^, we utilized different databases to conduct a detailed analysis of STEAP4 expression in HNSCC. The ENCORI database, which statistically analyzed and calibrated data from 44 normal and 502 HNSCC samples, revealed a more pronounced downregulation of STEAP4 in HNSCC (Figure 2A). Another tumor-related database, UALCAN portal, also confirmed this result (Figure 2B). Notably, the expression of STEAP4 in HNSCC tended to be decreased in advanced tumor stages (Figure 2C).

To further investigate the potential role of low STEAP4 expression in HNSCC, we assessed prognostic information, including OS, DFS, and RFS in HNSCC patients. One-way Cox regression analysis indicated that STEAP4 acted as a protective factor in head and neck cancer (HNC). The OS of HNC patients with low expression of STEAP4 showed a downward trend, although the difference was not statistically significant (*p* = 6.02e-02) (Figure 2D). This may be due to the small sample size applied for survival analysis. To delve deeper, we explored the GSE2837 dataset related to HNSCC in HNC, and statistically analyzed 40 samples from 28 HNSCC patients. Interestingly, different probes yielded varying analytical results, although none of the results were statistically significant (*p* = 0.279249, and *p* = 0.985529). In this dataset, STEAP4 was identified to serve as a protective factor (HR = 0.30) or a risk factor (HR = 1.02) for HNSCC, as shown in the RFS (Figure 2E). At this point, the results remain somewhat contentious.

Upon the analysis in OShnscc, it is discovered that in dataset GSE31056, STEAP4 was a risk factor (HR = 3.9889) that predicted poor prognosis in patients (*p* < 0.0001). TCGA and other datasets GSE65858 also predicted STEAP4 as a risk factor, except for datasets GSE3292 (Figure 2F, Table 2). The result in GSE31056 and TCGA was further confirmed in the GSE27020 dataset (HR = 2.1358, *p* = 0.0366) (Figure 2G). HNSCC, a specific type of cancer, encompasses OSCC, oropharyngeal squamous cell carcinoma (OPSCC), laryngeal squamous cell carcinoma (LSCC), and nasal and nasopharyngeal squamous cell carcinoma^28^. So, the contradictory above-mentioned outcomes may be attributed to differences in sample composition. Among these, STEAP4 acted as an independent risk factor in OSCC (GSE31056) and LSCC (GSE27020), and its high expression was positively associated with shorter OS and RFS in these patients (Figure 2F-G). This indicates that OSCC and LSCC exhibit unique tumor heterogeneity within HNSC, potentially reflecting distinct tumor characteristics and gene expression profiles.

### STEAP4 is regarded as a risk factor in OSCC accompanied by worse prognosis

**Figure 3:**
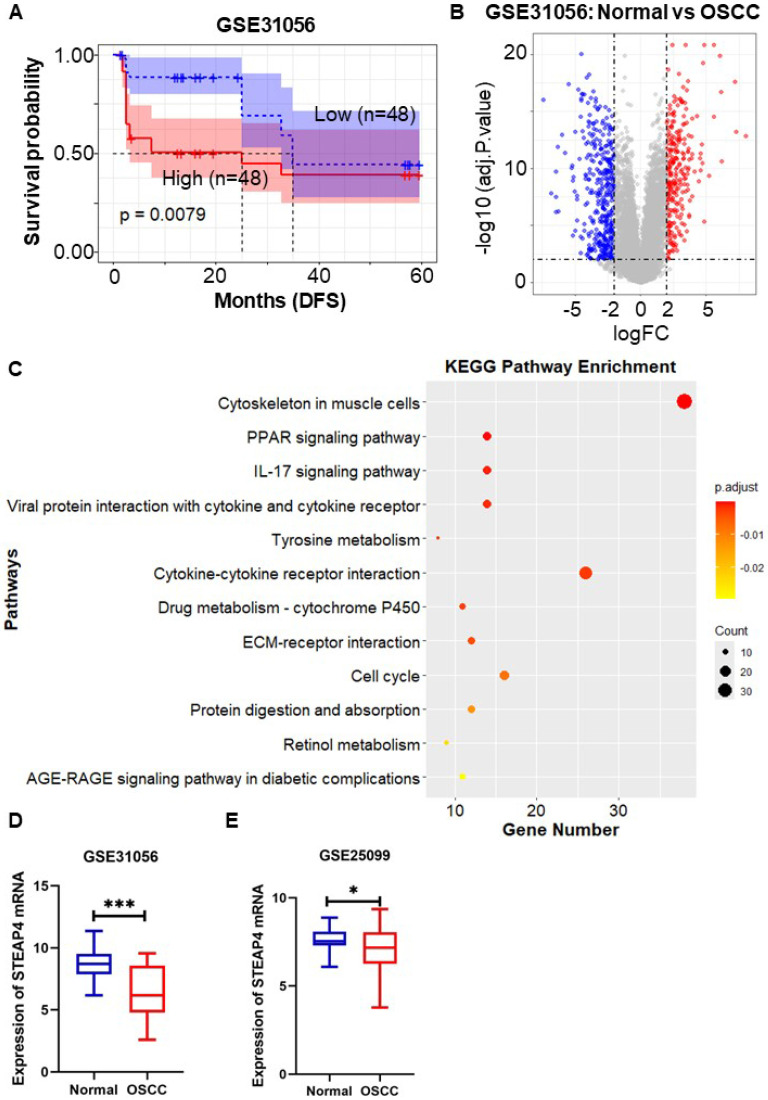
** Role of STEAP4 in OSCC.** (A) DFS for STEAP4 levels for patients with HNSCC in GSE31056. (B) Volcano plot of DEGs in GSE31056 (p < 0.01, |logFC| = 2). (C) KEGG pathway enrichment analysis of DEGs. (D & E) Expression of STEAP4 in GSE31056 (D) and GSE25099 (E).

To validate the aforementioned findings, we utilized the OSCC-related dataset GSE31056 to evaluate the survival and prognostic significance of STEAP4, utilizing the Kaplan-Meier method implemented in the R language through the survival package. The dataset comprised 96 tissue samples from 24 patients, encompassing histologically normal tissues, OSCC tissues, and normal adjacent margin tissues. The Kaplan-Meier survival curves depicted the impact of STEAP4 expression on RFS in OSCC patients. Consistent with previous findings, our results indicated that high expression of STEAP4 was associated with decreased RFS (*p* = 0.0079) (Figure 3A), suggesting that STEAP4 may function as a risk factor in OSCC. The finding indicates that STEAP4 exhibits elevated expression in OSCC, functioning as a potential oncogene.

In order to ascertain the elevated expression of STEAP4 in OSCC, we conducted an analysis of STEAP4 expression in the GEO dataset GSE31056. This analysis identified 682 DEGs from the 96 tissue samples, comprising 277 up-regulated genes and 405 down-regulated genes (Figure 3B). Clustering of DEGs based on functional and signaling pathway analysis, as well as KEGG and GO enrichment analyses, revealed enrichment in external encapsulating structure organization (BP), collagen-containing extracellular matrix (CC), receptor ligand activity (MF), and cytoskeleton in muscle cells, as depicted in Figure 3C and Supplementary Figure 1. Notably, STEAP4 was identified as a down-regulated gene among the DEGs, indicating that its expression was lower in OSCC compared to adjacent normal tissues (Figure 3D), contradicting our initial conjecture. Subsequently, we further investigated the expression of STEAP4 in different OSCC datasets, which revealed that STEAP4 was expressed slightly lower in OSCC than in normal tissues, although the difference was not statistically significant (Figure 3E).

### Mechanism of tumor heterogeneity of STEAP4 in OSCC

**Figure 4:**
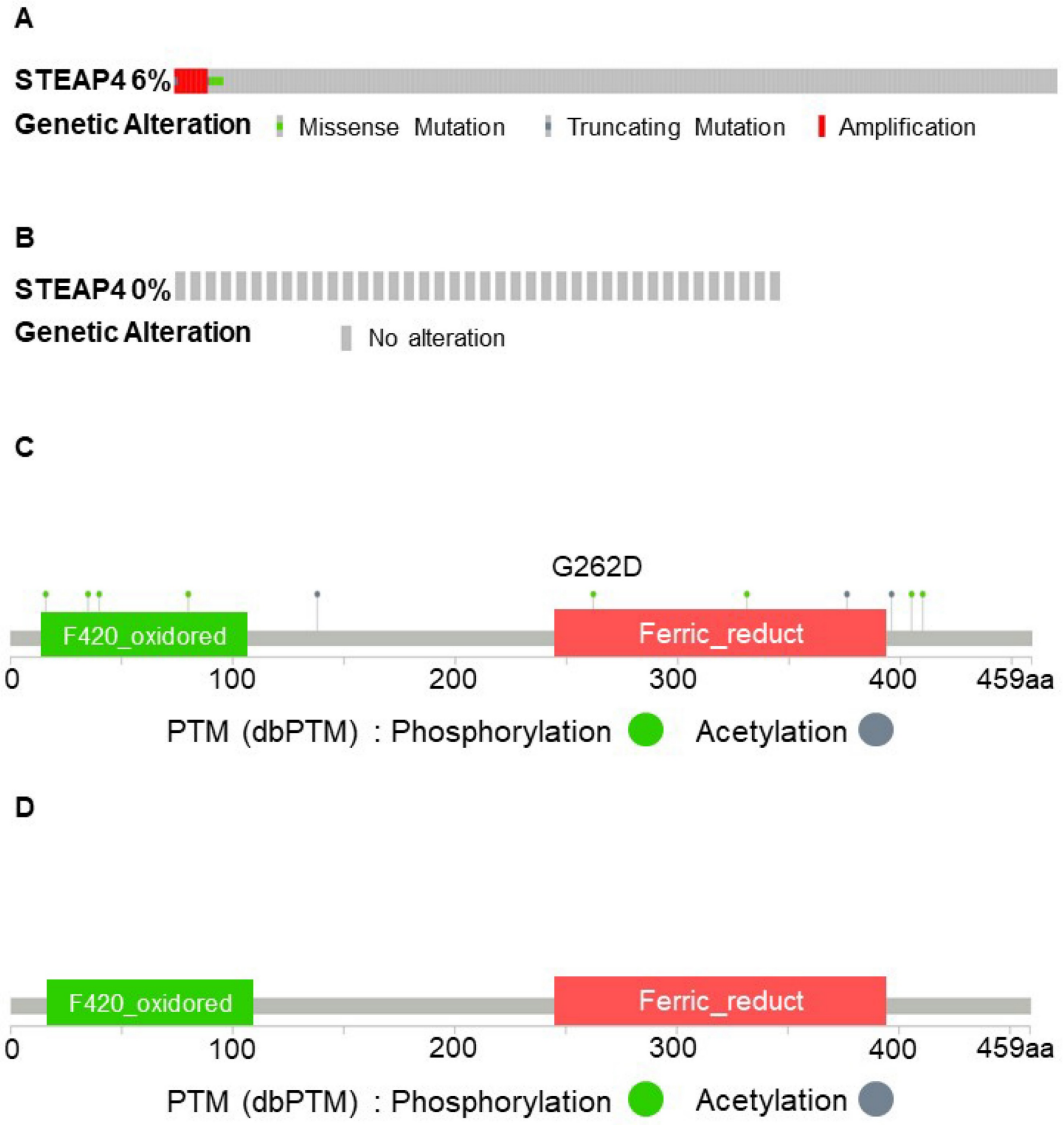
** Mutation of STEAP4 in HNSCC and OSCC patients.** (A & B) Comparison of mutation percentage and types of STEAP4 in HNSCC (A) and OSCC (B) patients from The Cancer Genome Atlas, PanCancer Atlas. (C & D) Two-dimensional structure of the STEAP4 protein, showing the STEAP4 mutation sites in HNSCC (C) and OSCC (D).

To elucidate the mechanisms underlying the unique expression pattern of STEAP4 in OSCC, we conducted an analysis of genetic alterations in STEAP4 across different datasets within cBioPortal. Varying degrees of gene amplification in HNSCC, accompanied by missense and truncating mutations in most patients across different TCGA cohorts (Figure 4A-B). Interestingly, almost all OSCC patients did not exhibit any mutations in the STEAP4 gene. This led us to speculate that the lack of significant differences in STEAP4 mRNA levels between normal and tumor tissues in OSCC could be attributed to genetic copy number alterations at the transcriptional level (Figure 4C-D). In other words, compared to HNSCC, STEAP4 underwent transcriptional-level mutations, which could potentially explain its transition from a protective factor in HNSCC to a risk factor in OSCC. However, further studies are required to supplement and confirm this conjecture.

**Figure 5:**
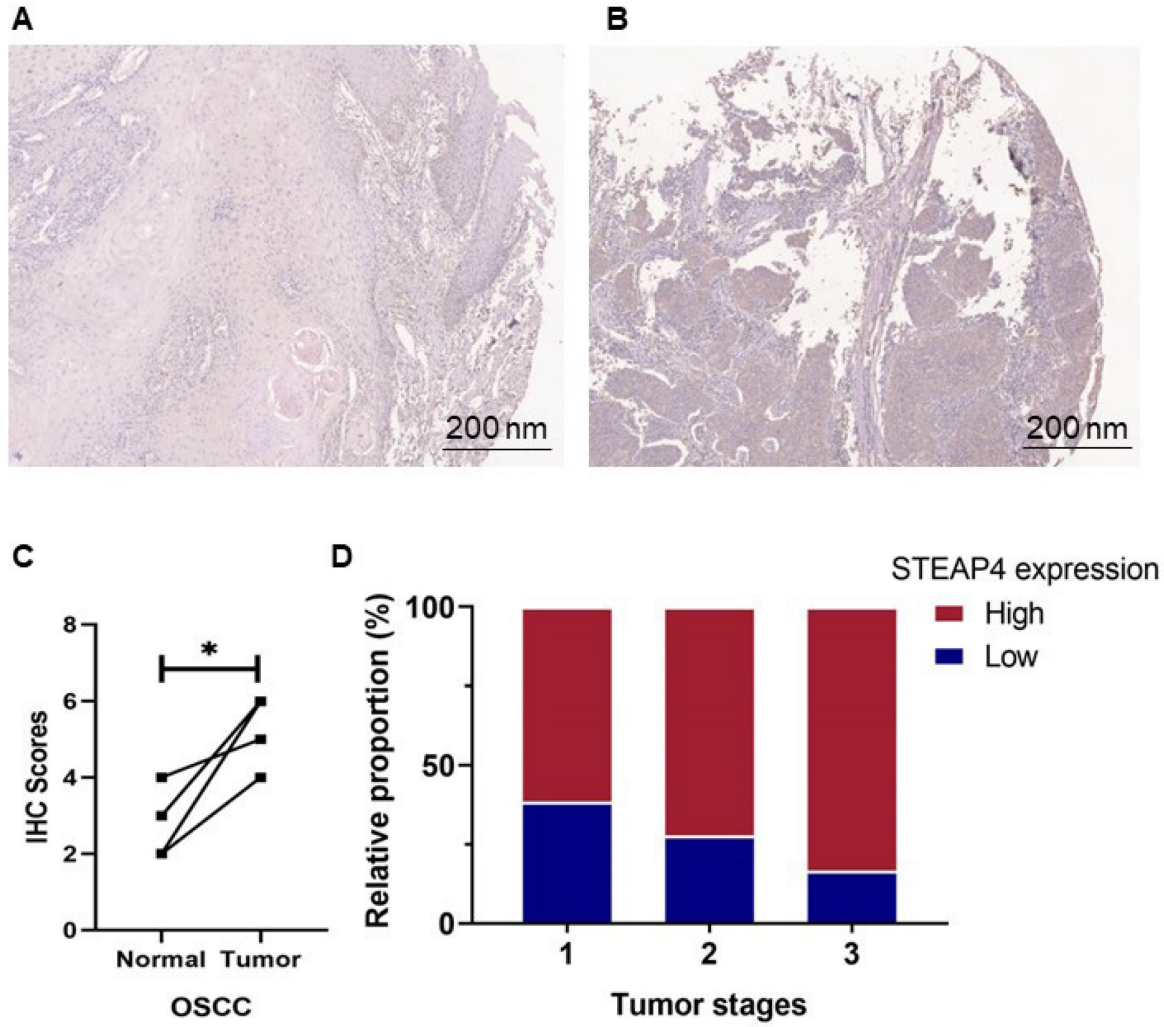
** Expression level of STEAP4 at the protein level.** (A & B) Representative staining of STEAP4 in normal (A) and OSCC (B) tissues. (C) IHC scores between normal tissues and tumor (p < 0.05). (D) Proportion of Tumor stages with increasing expression level of STEAP4 in OSCC patients.

To further investigate the expression and distribution of STEAP4 in the oral cavity, we performed immunohistochemical staining and scoring on OSCC tissues. These samples included 14 with low expression and 30 with high expression of STEAP4, with 4 pairs of corresponding adjacent tissues and cancers. Contrary to mRNA predictions, immunohistochemical staining revealed higher expression levels of STEAP4 in OSCC tissues compared to adjacent normal tissues (Figure 5A-B). Notably, the staining levels of STEAP4 were elevated in cancerous tissues compared to the corresponding adjacent tissues in the four cases (Figure 5C). Clinicopathological analysis showed no statistically significant differences in STEAP4 expression based on gender, age group, tumor site, or various clinical stages. However, the majority of OSCC patients developed tumors in the tongue, with 84.6% of these patients exhibiting high expression of STEAP4 (Table 3). Furthermore, high protein levels of STEAP4 were more common in patients with advanced disease (Figure 5D). These findings suggest that high protein levels of STEAP4 are indicative of a poor prognosis for patients, contradicting the mRNA predictions in the dataset. We hypothesize that this discrepancy may be attributed to systematic biases arising from individual differences in clinical samples or the insufficient sample size of the mRNA dataset, which may not fully reflect the overall picture due to the limited number of OSCC-related studies.

Consistent with these results, the protein expression level scoring of STEAP4 on the Bgee website demonstrated elevated expression on the surface and in the tongue, with scores of 84.98 and 80.74, respectively (Supplementary Figure 2). This aligns with the clinicopathological parameters analyzed earlier. Additionally, recent studies have shown that approximately 40% of OSCC cases occur in the floor of the mouth, lateral margins of the tongue, or the ventral tongue^29^. Remarkably, in concordance with the immunohistochemical staining results of clinical tissues, we unexpectedly found that normal tongue squamous epithelial cells lacked STEAP4 expression. This phenomenon suggests considerable variation in STEAP4 expression among different oral mucosal tissues, which may contribute to the tumor heterogeneity of STEAP4 in OSCC. These findings not only shed light on the potential molecular mechanisms underlying the presence of STEAP4 in OSCC, but also provide a new direction for the detection and diagnosis of OSCC.

## Discussion

### Origin and latest research on STEAP4

The STEAP family of metalloproteinases, involved in iron and copper homeostasis and various cellular processes, contains five known homologs, STEAP1, STEAP1B, STEAP2, STEAP3, and STEAP4^1^,^30^-^32^. In mammals, STEAP1-4 also regulate cell proliferation, apoptosis, attenuate oxidative stress, and mediate the transferrin cycle^5^,^33^.

STEAP1, a protein of 339 amino acids (39.9 kDa), does not independently exhibit Fe(3+) or Cu(2+) reductase activity due to the absence of an N-terminal NADPH-binding FNO structural domain^4^,^21^,^34^. STEAP1 localizes to cell membranes and is thought to be involved in tight junctions, gap junctions, or cell adhesion, acting as an ion channel or transporter protein, which also facilitates intercellular communication. Its overexpression in cancers may promote cancer cell proliferation and invasion by regulating ion and small molecule concentrations^35^,^36^. Blocking STEAP1 with a specific monoclonal antibody in LNCaP cells increased cell death, suggesting that STEAP1 may promote cancer cell proliferation or prevent apoptosis^35^. As a STEAP1 homologue, STEAP1B also lacks Fe(3+) or Cu(2+) reductase activities, but is distinguished by having two transcripts, STEAP1B1 and STEAP1B2^30^. STEAP1B1 mRNA and STEAP1B2 mRNA are highly expressed in benign prostate cell lines and androgen-dependent PCa cell lines, respectively^30^.

STEAP2, known as six-transmembrane protein of prostate 1 (STAMP1), possesses an N-terminal NADPH-binding FNO structural domain, giving it iron-copper reductase properties^4^,^5^. Highly expressed in the Golgi complex, trans-Golgi network, and plasma membranes^31^,^37^, STEAP2 may act as an endogenous or exogenous receptor, such as lipids and proteins, or as a regulator of protein transport and sorting mechanisms^31^. *In vitro* and *in vivo* studies have shown that STEAP2 regulates genes involved in the cell cycle, leading to partial cell cycle arrest in the G0-G1 phase. Knockdown of STEAP2 promotes apoptosis in prostate cancer cells, increasing prostate cancer cell proliferation^38^.

STEAP3, the unique tumor suppressor in the STEAP family in PCa progression, is involved in transferrin endosomal-mediated iron uptake, which mediates cell death^39^,^40^. It participates in apoptosis and cell cycle regulation by interacting with pro-apoptotic factors Nix and Myt1 kinase^41^. Additionally, STEAP3 mediates the secretion of the translationally controlled tumor protein (TCTP)^42^, a Ca2þ and microtubule-binding protein implicated in cell cycle progression and malignant transformation^43^, thereby acquiring antiapoptotic activities 44,45. STEAP3-induced TCTP secretion may also sensitize cancer cells to apoptosis^46^.

STEAP4, the latest member of the family, is involved in inflammatory responses, fatty acid metabolism, and glucose metabolism^2^,^47^. Overexpression of STEAP4 was initially discovered in white and brown adipose tissues and found to be strongly induced by growth hormone^48^, TNF-α^3^, interleukin-6 (IL-6)^49^ and interleukin-1b (IL-1b)^50^. Subsequent research has revealed that STEAP4 also plays a crucial role in promoting adipocyte proliferation and apoptosis. Qin *et al*. conducted a study demonstrating that treatment of preadipocytes with antibodies against STEAP4 resulted in the emergence of apoptotic morphology, followed by mitochondrial damage, leading to cellular swelling and pyrolysis^51^. Similarly, treatment of adipocytes with antibodies against STEAP4 reduced the rate of cell proliferation and the number of cells entering the S-phase of the cell cycle^51^. The inflammatory cytokine interleukin 17 (IL17) increases intracellular copper by inducing STEAP4 in CRC, leading to the activation of X-linked inhibitor of apoptosis (XIAP), which inhibits CASP3 activity^52^. Furthermore, GO annotation analysis indicated that STEAP4 is associated with biological pathways, such as fat cell differentiation (Table 1). These studies have explored the association of STEAP4 with obesity, insulin resistance, inflammation, and prostate cancer progression^51^,^53^.

Previous research has shown that STEAP4 is abundantly expressed in prostate cancer cells in the Golgi complex and plasma membrane, with aberrant expression predicting it plays an essential role in the development of prostate cancer 54. STEAP4 may cause the generation of reactive oxygen species (ROS) through its oxidoreductase activity, leading to the expression of transcription factor ATF4, which promotes the growth and progression of prostate cancer, eventually leading to chemoresistance^55^. ATF4 is a transcription factor involved in tumorigenesis that can be induced by ER, metabolic, oxidative, and other stresses^56^,^57^. Furthermore, STEAP4 participates in promoting an LPS-induced inflammatory microenvironment. LPS increases STEAP4 expression through the cGMP-PKG pathway, promoting PCa proliferation^58^. PPI networking revealed that STEAP4 may exert biological effects through the KLK family. The KLK family was initially discovered in the pancreas by Kraut *et al.* in 1930, and this family genes are mainly located on chromosome 19q13.3 to 13.4. These genes are involved in the regulation of physiological functions in cardiovascular, renal, and neurological systems, among others^59^,^60^. The KLK family consists of 15 members, with KLK1, KLK2, and KLK3 identified as potential biotherapeutic targets for prostate cancer^23^,^61^. KLK3, also known as prostate-specific antigen (PSA), has been widely used in clinical settings for many years. Additionally, KLK4 has shown biotherapeutic potential in ovarian cancers and TNBCs 24,25, and KLK5 has been associated with the prognosis of patients with CRCs 26. However, its effect on STEAP4 remains untested.

Recent studies have shown that the STEAP family is involved in processes such as iron and copper uptake and transport, regulation of apoptosis and cell cycle, playing crucial roles in the development, progression and metastasis of different types of cancer^27^. During the development of PCa, all STEAP members except STEAP3 tend to be overexpressed^4^. In other types of tumors, the expression of STEAP family members varies^27^. Research has shown that STEAP4 not only plays a role in the development and progression of breast cancers 62,63, but also participates in the regulation of inflammatory responses in CRC^54^. Our previous research demonstrated that the expression of STEAP4 is lower in CRC tissues compared to normal tissues, and this expression is positively correlated with immune-related biomarkers^27^. Conversely, STEAP4 has been shown to act as an oncogene in gastric cancer, and is strongly associated with poor prognosis in patients^64^. Notably, STEAP4 has rarely been reported in other cancer types, such as BRCA, HNSCC, and OSCC.

### Current status and research progress of OSCC

Oral cancer, the most common malignant tumor of the head and neck (excluding non-melanoma skin tumors), has a high global incidence^65^,^66^ and a low 5-year survival rate^67^. Recent studies have shown that the expression levels of specific biomarkers IL-8, IL-6, and TNF-α in saliva are associated with signaling pathways in the development of various oral lesions and oral cancers^68^. Reports indicate that circadian rhythms significantly affect salivary flow rate, salivary composition, and the expression of cytokines and regulatory factors in oral cancers.

OSCC, the most common type of oral cancers, accounts for about 90% of oral cancer cases^69^. The 5-year survival rate for OSCC is only 41%, with an annual improvement rate of less than 1%^70^. Major risk factors for OSCC include smoking, heavy alcohol consumption^71^, and the presence of OPMDs, such as oral proliferative leukoplakia (PL)^72^,^73^. Despite the availability of diagnostic criteria for oral precancerous lesions and the latest clinical grading for oral cancer diagnosis (AJCC 8th edition staging), OSCC diagnosis has high intra- and inter-observer variability^74^,^75^, making it challenging to predict the progression of precancerous lesions to OSCC accurately^76^. Therefore, more accurate biomarkers for predicting early OSCC lesions and cancer progression would enhance OSCC diagnosis and treatment targeting^76^.

Several studies have reported biomarkers contributing to OSCC diagnosis and prognosis. Li *et al.* proposed a diagnostic method using mRNAs of certain genes as biomarkers, detecting increased expression of markers, such as DUSP1, H3F3A, IL1B, IL8, OAZ1, S100P, and SAT, with high sensitivity and specificity in OSCC subjects^77^. Studies have also indicated that miRNAs may play a key role as promising salivary biomarkers with high sensitivity and specificity, and minimally invasive advantages in OSCC diagnosis^78^. However, the diagnostic performance of miR-136 in OSCC remains inconsistent in reported studies. Liu *et al*. reported the accuracy of salivary miR-136, miR-27B, and miR-27b for OSCC diagnosis as high as 80.0%, 77.0%, and 78.0%, respectively^67^. Recent research advances have expanded the understanding of OSCC tumorigenesis and progression by analyzing biomarkers predicting OSCC progression from multiple aspects, including genomics, proteomics, metabolomics, immunomics, and microbiomics, proposing that composite biomarkers discovered through multi-omics integration will enhance the reliability, sensitivity, and specificity of OSCC diagnostic methods and therapeutic regimens^79^.

### Current progress and deficiencies in the research

To date, our team has revealed the potential of STEAP4 as a prognostic biomarker in CRC^27^ and GC^64^. In this study, based on TCGA and GEO databases, we used publicly available online analytical tools and bioinformatics software, such as "R language", for data mining. We discovered that STEAP4, as a metalloreductase, exhibits unique tumor heterogeneity in different cancer types and subtypes. PPI network interaction analysis showed that STEAP4 closely interacts with the KLK family, implying that the KLK family may influence STEAP4's role in cancer.

More importantly, we analyzed HNSCC-associated (GSE2837) and OSCC-associated (GSE31056) GEO datasets, finding that STEAP4 mRNA expression was reduced in both HNSCC and OSCC. However, KM survival curve analysis and univariate COX analysis indicated that STEAP4 was a protective factor in HNSCC, but a risk factor in OSCC. We collected clinical tissue samples from OSCC patients for further analysis. Immunohistochemical staining evaluation showed elevated STEAP4 protein levels in OSCC. Clinicopathological parameters indicated that the tongue is the primary site in most OSCC patients, with high STEAP4 levels closely associated with advanced recurrence and metastasis. According to the AJCC 7th edition clinical staging of oral cancers, STEAP4 expression was higher in stage 3 and above. Hereby, we conjectured that, aside from the small sample size of OSCC in datasets and individual differences in clinical organization, genetic mutations and histological differences within the oral cavity between HNSCC and OSCC may account for this inconsistency.

Genetic alterations have been reported to cause aberrant activation of oncogenic pathways in OSCC, such as EGFR^80^, Wnt/β-catenin^81^, JAK/STAT^82^, NOTCH^83^, PI3K/AKT/mTOR^84^, MET^85^, and RAS/RAF/MAPK, as well as blockade of anticancer pathways, such as TP53/RB^86^and pl6/Rb/cyclin D1^87^. These pathways play crucial roles in OSCC development. However, our current investigation found that STEAP4 was almost not mutated in OSCC, while different degrees of gene copy number variation, such as gene amplification and deletion, occur in HNSCC. Therefore, we conjectured that gene mutations in STEAP4 might be the main reason for STEAP4 heterogeneity in HNSCC and OSCC. In summary, our research verified that STEAP4 exhibits tumor heterogeneity in HNSCC and OSCC and can serve as an independent predictor for assessing the prognosis of OSCC patients.

## Supplementary Material

Supplementary figures and tables.

## Figures and Tables

**Table 1:** GO annotations of STEAP4 in UniProt.

ASPECT	TERM
Cellular Component (CC)	early endosome membrane
	endosome
	extracellular exosome
	Golgi membrane
	membrane
	nucleoplasm
	plasma membrane
Molecular Function (MF)	cupric reductase activity
	electron transfer activity
	FAD binding
	ferric-chelate reductase (NADPH) activity
	heme binding
	metal ion binding
Biological Process (BP)	copper ion import
	fat cell differentiation
	iron import into cells
	protein homotrimerization

**Table 2:** Univariate Cox regression analysis of STEAP4 expression with OS in each OShnscc dataset

Dataset	p-value	HR	95%CI
**GSE31056**	< 0.0001	3.9889	2.0504 ~ 7.7600
**GSE3292**	0.4529	0.4482	0.0551 ~ 3.6439
**TCGA**	0.7446	1.0526	0.7733 ~ 1.4329
**GSE65858**	0.7452	1.0815	0.6743 ~ 1.7347

**Table 3:** Relationship between STEAP4 protein level and clinicopathological parameters of OSCC patients.

Clinicopathological parameters	STEAP4 expression		Total	*P*-value
	Low	High		
**Gender**			44	0.679
Male	7 (29.2%)	17 (70.8%)	24	
Female	7 (35.0%)	13 (65.0%)	20	
**Age** (**years**)				0.087
≤ 64	5 (20.8%)	19 (79.2%)	24	
> 64	9 (45.0%)	11 (55.0%)	20	
**Tumor site**			44	0.378
Lip	2 (50.0%)	2 (50.0%)	4	
Palate	3 (37.5%)	5 (62.5%)	8	
Buccal	6 (46.2%)	7 (53.8%)	13	
Tongue	2 (15.4%)	11 (84.6%)	13	
Teeth and periodontal tissues	1 (16.7%)	5 (83.3%)	6	
**T**			37	0.607
T1	5 (38.5%)	8 (61.5%)	13	
T2	5 (27.8%)	13 (72.2%)	18	
T3	1 (16.7%)	5 (83.3%)	6	
**N**			27	0.302
N0	6 (31.6%)	13 (68.4%)	19	
N1-3	1 (12.5%)	7 (87.5%)	8	
**AJCC stage**			38	0.479
I	5 (38.5%)	8 (61.5%)	13	
II	4 (30.8%)	9 (69.2%)	13	
III	2 (16.7%)	10 (83.3%)	12	
**Lymph node invasion**			27	0.302
Negative	6 (31.6%)	13 (68.4%)	19	
Positive	1 (12.5%)	7 (87.5%)	8	

AJCC: American Joint Committee on Cancer; N: Node; T: Tumor.
