# Supplementary material for: Tumor Heterogeneity of STEAP4 in Malignant Progression of Oral Squamous Cell Carcinoma
Source: J Cancer. 2024 Nov 4;15(20):6754–67. doi: 10.7150/jca.101470 (PMC11632996; doi:10.7150/jca.101470)
Supplement: Supplementary file 1 — Supplementary figures and tables. [file jcav15p6754s1.pdf]

Supplementary Table 1. Expression levels of STEAP4 in different organs.

	**STEAP4 expression**	
**Organism part**	**RNA**	**Protein**
adipose	++	N.E.
adipose tissue	++	+++
adrenal	++	N.E.
adrenal gland	++	+++
amygdala	+	+
animal ovary	+	+
aorta	++	+++
aortic valve	N.E.	+++
appendix	++	N.E.
artery	++	N.E.
atrial septum	N.E.	+++
atrium auricular region	++	N.E.
B cell	N.E.	-
basal ganglion	-	N.E.
bladder	++	N.E.
blood	++	N.E.
bone marrow	++	+++
brain	+	++
brain fragment	-	N.E.
brain meninx	+	N.E.
breast	++	N.E.
Brodmann (1909) area 24	-	N.E.
Brodmann (1909) area 9	-	N.E.
C1 segment of cervical spinal cord	+	N.E.
caudate nucleus	+	N.E.
CD4-positive T cell	N.E.	-
CD8-positive T cell	N.E.	-
cerebellar hemisphere	+	N.E.
cerebellum	+	N.E.
cerebral cortex	-	N.E.
choroid plexus	+	N.E.
colon	++	+++
coronary artery	++	N.E.
cortex of kidney	+	N.E.
diaphragm	++	N.E.
diencephalon	-	N.E.
dorsal thalamus	+	N.E.
duodenum	+	+++
dura mater	++	N.E.
EBV-transformed lymphocyte	-	N.E.

ectocervix	++	N.E.
endocervix	++	N.E.
endometrium	++	+++
epididymis	++	N.E.
esophagogastric junction	++	N.E.
esophagus	++	+++
esophagus mucosa	++	N.E.
esophagus muscularis mucosa	++	N.E.
eye	++	N.E.
fallopian tube	++	+++
forebrain	-	N.E.
forebrain and midbrain	-	N.E.
frontal cortex	N.E.	-
frontal lobe	-	N.E.
gall bladder	++	-
gallbladder	N.E.	+++
globus pallidus	+	N.E.
greater omentum	++	N.E.
heart	++	+++
heart left ventricle	++	-
hindbrain	-	N.E.
hippocampal formation	+	N.E.
hippocampus proper	-	N.E.
hypothalamus	-	N.E.
inferior vena cava	N.E.	+++
kidney	++	+++
large intestine	+	N.E.
left atrium	N.E.	+++
left cardiac atrium	++	++
left kidney	+	N.E.
left renal cortex	+	N.E.
left renal pelvis	+	N.E.
left ventricle	N.E.	+++
leukocyte	++	N.E.
liver	++	+++
locus ceruleus	+	N.E.
lower leg skin	++	N.E.
lung	++	+++
lymph node	++	+++
medulla oblongata	++	N.E.
midbrain	-	N.E.
middle frontal gyrus	+	N.E.
middle temporal gyrus	+	N.E.
minor salivary gland	++	N.E.

mitral valve	++	+++
monocyte	N.E.	-
muscle of arm	+	N.E.
muscle of leg	+	N.E.
natural killer cell	N.E.	-
nucleus accumbens	-	N.E.
occipital cortex	+	N.E.
occipital lobe	-	N.E.
olfactory apparatus	+	N.E.
ovary	++	+++
pancreas	++	+++
parietal lobe	+	N.E.
parotid gland	++	N.E.
penis	++	N.E.
pineal body	++	N.E.
pituitary gland	++	+++
placenta	++	+++
platelet	N.E.	-
prefrontal cortex	-	N.E.
prostate	++	+++
prostate gland	++	-
pulmonary artery	N.E.	+++
pulmonary valve	++	+++
pulmonary vein	N.E.	+++
putamen	+	N.E.
rectum	++	+++
renal pelvis	-	N.E.
retina	N.E.	-
right atrium	N.E.	+++
right cardiac atrium	N.E.	++
right renal cortex	-	N.E.
right renal pelvis	-	N.E.
right ventricle	N.E.	+++
saliva-secreting gland	+	N.E.
salivary gland	+	+++
seminal vesicle	++	N.E.
sigmoid colon	++	N.E.
skeletal muscle	++	N.E.
skeletal muscle of trunk	+	N.E.
skeletal muscle tissue	+	N.E.
skin	+	N.E.
small intestine	+	+++
small intestine Peyer's patch	+	N.E.
smooth muscle	N.E.	+++

smooth muscle tissue	++	N.E.
spinal cord	+	-
spleen	++	+++
stomach	+	+++
subcutaneous adipose tissue	++	N.E.
submandibular gland	++	N.E.
substantia nigra	+	-
suprapubic skin	++	N.E.
telencephalon	-	N.E.
temporal lobe	-	N.E.
testis	++	+++
throat	++	N.E.
thymus	-	N.E.
thyroid	++	+++
thyroid gland	++	N.E.
tibial artery	++	N.E.
tibial nerve	++	N.E.
tongue	++	N.E.
tonsil	+	+++
trachea	++	N.E.
transformed skin fibroblast	++	N.E.
transverse colon	+	N.E.
tricuspid valve	++	+++
umbilical cord	++	N.E.
urinary bladder	++	+++
uterine cervix	++	N.E.
uterus	++	N.E.
vagina	++	N.E.
vas deferens	++	N.E.
ventricular septum	N.E.	+++
vermiform appendix	++	+++
zone of skin	++	N.E.

*** N.E.: Not Examined**

Supplementary Table 2. Expression levels of STEAP4 in diseases.

	STEAP4 expression
**Diseases**	**RNA**
adenovirus infection	++
astrocytoma	+
B-cell non-Hodgkin lymphoma	+
bladder transitional cell carcinoma	+
breast adenocarcinoma	+
breast tumor luminal	+++
cervical adenocarcinoma	++
cervical squamous cell carcinoma	++
cholangiocarcinoma	+
chromophobe renal cell carcinoma	+
chronic lymphocytic leukemia	-
colon adenocarcinoma	-
colon mucinous adenocarcinoma	+++
colorectal adenocarcinoma	+
endometrial adenocarcinoma	+
escherichia coli infection	++
esophageal adenocarcinoma	++
follicular thyroid carcinoma	++
gastric adenocarcinoma	+
glioblastoma multiforme	-
glioma	-
group A streptococcal infection	++
head and neck squamous cell carcinoma	+
hepatocellular carcinoma	+
HER2 Positive Breast Carcinoma	++
influenza	++
invasive lobular carcinoma	+
juvenile idiopathic arthritis	++
Kawasaki disease	++
lung adenocarcinoma	++
lymphoma	+
malaria	++
melanoma	+
meningococcal infection	++
normal - adjacent to bladder transitional cell carcinoma	+
normal - adjacent to breast adenocarcinoma	++
normal - adjacent to cholangiocarcinoma	++
normal - adjacent to chromophobe renal cell carcinoma	+
normal - adjacent to endometrial adenocarcinoma	+
normal - adjacent to follicular thyroid carcinoma	++

normal - adjacent to gastric adenocarcinoma	+
normal - adjacent to head and neck squamous cell carcinoma	++
normal - adjacent to hepatocellular carcinoma	++
normal - adjacent to lung adenocarcinoma	++
normal - adjacent to prostate adenocarcinoma	++
normal - adjacent to renal cell carcinoma	+
normal - adjacent to squamous cell lung carcinoma	++
normal - amygdala (GTEx)	+
normal - blood (GTEx)	++
normal - breast (GTEx)	++
normal - Brodmann (1909) area 24 (GTEx)	+
normal - Brodmann (1909) area 9 (GTEx)	+
normal - C1 segment of cervical spinal cord (GTEx)	+
normal - caudate nucleus (GTEx)	+
normal - cerebellar hemisphere (GTEx)	+
normal - cerebellum (GTEx)	+
normal - cerebral cortex (GTEx)	-
normal - cortex of kidney (GTEx)	+
normal - ectocervix (GTEx)	++
normal - endocervix (GTEx)	++
normal - esophagogastric junction (GTEx)	++
normal - esophagus mucosa (GTEx)	++
normal - esophagus muscularis mucosa (GTEx)	++
normal - hippocampus proper (GTEx)	+
normal - hypothalamus (GTEx)	+
normal - liver (GTEx)	++
normal - lower leg skin (GTEx)	++
normal - lung (GTEx)	++
normal - minor salivary gland (GTEx)	++
normal - nucleus accumbens (GTEx)	+
normal - ovary (GTEx)	+
normal - pancreas (GTEx)	+
normal - prostate gland (GTEx)	++
normal - putamen (GTEx)	+
normal - sigmoid colon (GTEx)	++
normal - skeletal muscle tissue (GTEx)	++
normal - stomach (GTEx)	++
normal - substantia nigra (GTEx)	+
normal - suprapubic skin (GTEx)	++
normal - thyroid gland (GTEx)	++
normal - transverse colon (GTEx)	+
normal - urinary bladder (GTEx)	++
normal - uterus (GTEx)	++
oligoastrocytoma	+

oligodendroglioma	+
ovarian adenocarcinoma	+
pancreatic adenocarcinoma	++
pneumococcal infection	++
prostate adenocarcinoma	++
renal cell carcinoma	+
Respiratory Syncytial Virus Infection	++
rhinovirus infection	++
sarcoma	++
squamous cell lung carcinoma	+
Staphylococcus aureus infection	++
triple-negative breast cancer	++
Tuberculosis	++

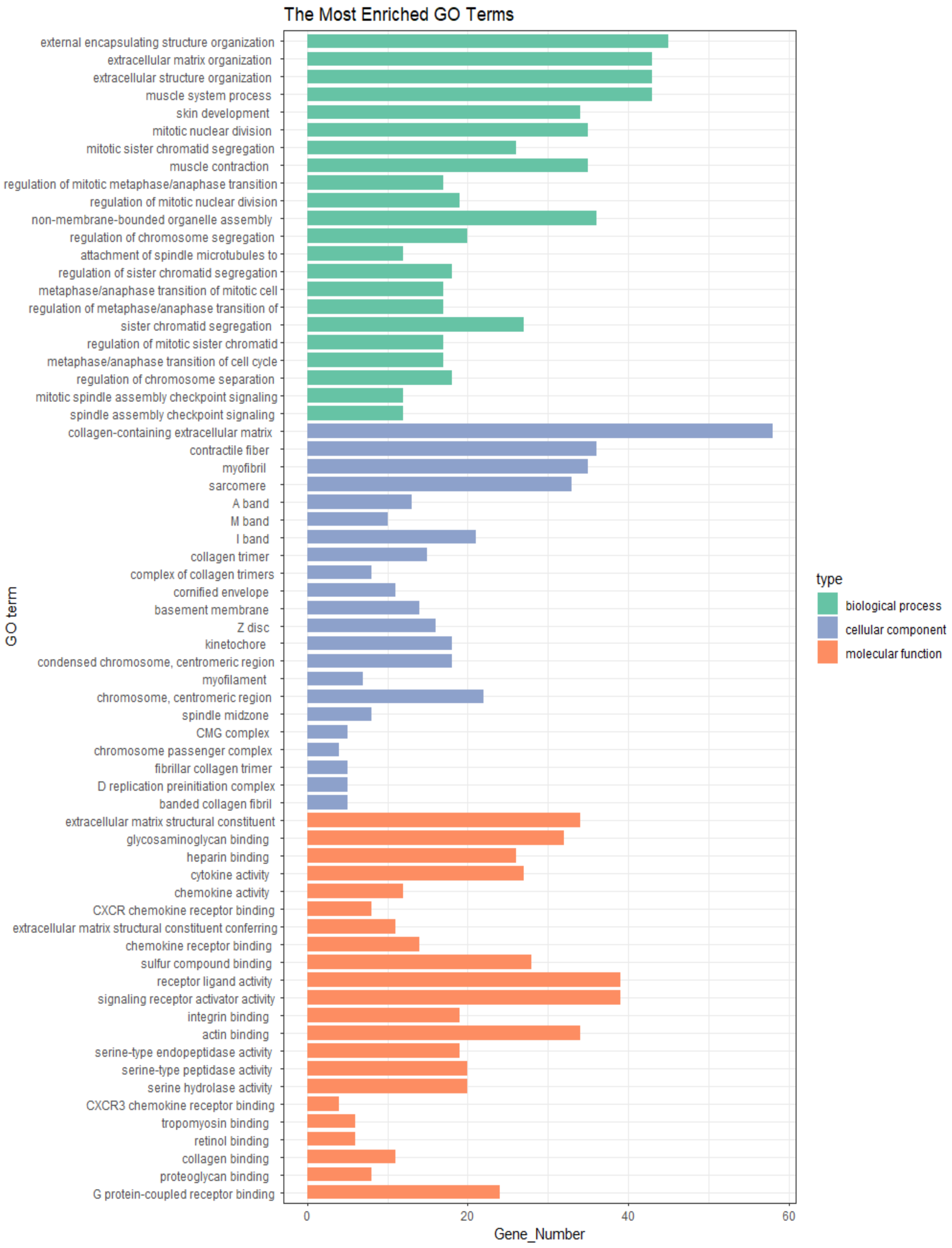

Supplementary Figure 1. GO enrichment analysis of DEGs.

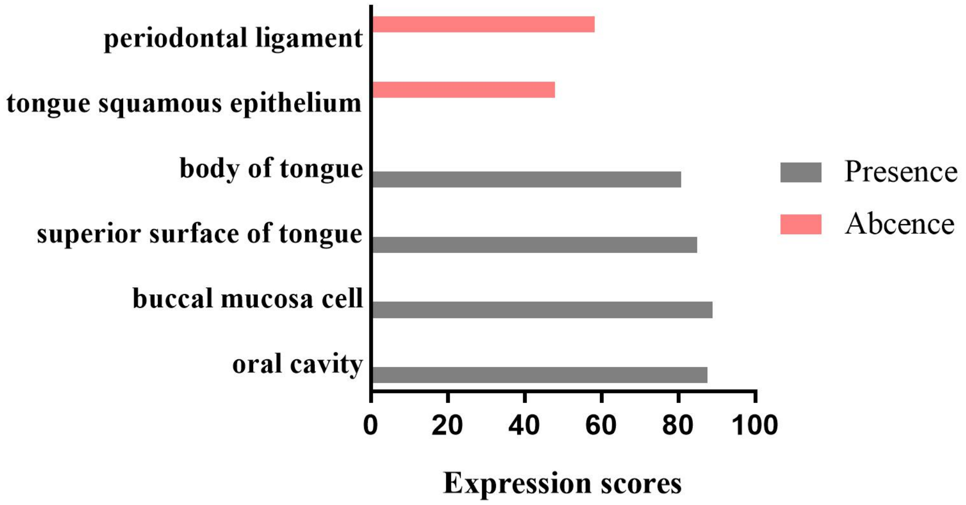

Supplementary Figure 2. Protein expression levels of STEAP4 in the Bgee website.
